# Answer to Dr. Shufeldt

**Published:** 1893-01

**Authors:** J. P. Turner

**Affiliations:** Veterinarian Sixth U. S. Cavalry; Fort Niobrara, Neb.


### COMMUNICATIONS.

### ANSWER TO DR. SHUFELDT.

EDITORS OF THE JOURNAL OF COMPARATIVE MEDICINE AND VETERINARY ARCHIVES:

In the December number, I notice an article from the pen of Dr. R. W. Shufeldt, a retired Medical Officer of the U. S. Army, in which he decries the general ignorance of equine anatomy, especially among Veterinarians, not only in civil but in military life.

To anyone who has closely studied the facilities for teaching anatomy in some of our veterinary colleges, the natural results are not surprising, especially where the veterinarian has learned his anatomy entirely from highly colored plates and Auzoux models, so that in many incidences must we agree with this gentleman in his conclusions.

However, Dr. Shufeldt intrudes into a territory, where one would think it extremely delicate for him to dwell in, when he criticises "the dense ignorance of anatomy prevailing among army Vetenarians and Cavalry Officers."

In this article, he makes use of the statement that "With the rarest exception, the Government veterinarian in the army knows little or nothing of the subject of equine anatomy and this stricture may with equal truth, be extended to include the West Point graduates in the Artillery and Cavalry Arms of the service."

Now, with one or two exceptions (relics of the "days of the empire" in the army), the Veterinary Corps of the U. S. Army consists of intelligent young men, who are graduates of the better class of Veterinary Schools, of this country and of England and who, from their complex duties as practitioners and teachers, are compelled to either keep abreast with the times in their technical studies or lose the respect of their respective regiments.

Under existing orders from the War Departments, Army Veterinarians act as instructors in Hippology to the class of Cavalry officers at their post, and any intelligent observer will readily see that an ignorant "quack," would not be tolerated by educated gentlemen, such as are our cavalry officers.

For many years, the army veterinarian has had a struggle for recognition, and in most of his efforts he has been handicapped by the precedent, which was set of formerly appointing any "mule-whacker," who was in hearty sympathy with the Colonel's profound ideas of Veterinary medicine, to the position of regimental veterinarian; but luckily, these days are past and we now see the army veterinarian in his proper place, both officially and socially, in many of our best cavalry regiments; while strenuous efforts are now being made by the War Department to have Congress increase the veterinary corps both in number, pay and rank.

Most unfortunately, Dr. Shufeldt has drawn his conclusions about the anatomical knowledge possessed by army Veterinarians and Cavalry Officers, from those graduated "mule-whackers" of a past decade, and from the class of Cavalry Officers, who would appoint such men to responsible positions; therefore we must accept his conclusions with due charity.

Would it be showing much wisdom and respect if I were to condemn the entire Medical Department of the U. S. Army, which department is one of the best organized military staffs in existence and consists of scientific and refined officers, simply because I have met officers of this corps who could talk far more intelligently about ornithology, botany and paleontology than about human anatomy and kindred medical subjects? Yet the two cases are parallel.

While it is a remarkable fact, yet it is nevertheless true, that the ordinary recently graduated West Pointer is comparatively ignorant of equine anatomy, yet these intelligent young officers cannot be blamed for this failing, since they receive no instruction whatever in this branch at the Military Academy.

These officers are given the best of instruction in artillery and cavalry tactics and manœuvres, yet of what good are all these elaborate studies to an officer unless he is acquainted with the secret of success in both branches of service, i. e., horses in perfect condition and training. Luckily for the cavalry officer, if he happens to be stationed at regimental headquarters, he can receive instruction each winter from the veterinarian.

For the artillery officer there is no such chance, since our Government fails to provide artillery with the services of a veterinarian.

The idea of establishing a Government school is a capital one and would be of inestimable value, both to the service and to the country at large.

While it yet seems an impossibility, we will hope that it will materialize in the near future. In conclusion, I beg leave to state that I think Dr. Shufeldt's article tends to do the cavalry officers of our army a great injustice, and is in extremely poor taste, since my observations show me that many of our younger officers are quite proficient in the study of equine anatomy and exhibit great zeal in learning this most difficult subject.

J. P. TURNER, V.M.D.,
Veterinarian Sixth U. S. Cavalry.

FORT NIOBRARA, NEB.

